# BoolFilter: an R package for estimation and identification of partially-observed Boolean dynamical systems

**DOI:** 10.1186/s12859-017-1886-3

**Published:** 2017-11-25

**Authors:** Levi D. Mcclenny, Mahdi Imani, Ulisses M. Braga-Neto

**Affiliations:** Electrical and Computer Engineering Department, College Station, Texas, USA

**Keywords:** Partially-Observed Boolean Dynamical Systems, Gene regulatory networks, Gene expression analysis, Boolean Kalman Filter, Particle filter, Network inference

## Abstract

**Background:**

Gene regulatory networks govern the function of key cellular processes, such as control of the cell cycle, response to stress, DNA repair mechanisms, and more. Boolean networks have been used successfully in modeling gene regulatory networks. In the Boolean network model, the transcriptional state of each gene is represented by 0 (inactive) or 1 (active), and the relationship among genes is represented by logical gates updated at discrete time points. However, the Boolean gene states are never observed directly, but only indirectly and incompletely through noisy measurements based on expression technologies such as cDNA microarrays, RNA-Seq, and cell imaging-based assays. The Partially-Observed Boolean Dynamical System (POBDS) signal model is distinct from other deterministic and stochastic Boolean network models in removing the requirement of a directly observable Boolean state vector and allowing uncertainty in the measurement process, addressing the scenario encountered in practice in transcriptomic analysis.

**Results:**

*BoolFilter* is an R package that implements the POBDS model and associated algorithms for state and parameter estimation. It allows the user to estimate the Boolean states, network topology, and measurement parameters from time series of transcriptomic data using exact and approximated (particle) filters, as well as simulate the transcriptomic data for a given Boolean network model. Some of its infrastructure, such as the network interface, is the same as in the previously published R package for Boolean Networks *BoolNet*, which enhances compatibility and user accessibility to the new package.

**Conclusions:**

We introduce the R package *BoolFilter* for Partially-Observed Boolean Dynamical Systems (POBDS). The *BoolFilter* package provides a useful toolbox for the bioinformatics community, with state-of-the-art algorithms for simulation of time series transcriptomic data as well as the inverse process of system identification from data obtained with various expression technologies such as cDNA microarrays, RNA-Seq, and cell imaging-based assays.

## Background

The Boolean Network (BN) model was introduced by Stuart Kauffman in a series of seminal papers [1–3]; see also [4]. This simple model has found extensive application in modeling cell biology processes involving regulatory networks of switching bistable components, such as the cell cycle process in *Drosophila* [5], *Saccharomyces cerevisiae* [6], and mammals [7]. The basic idea is that in a feedback biochemical network, based for example on the expression of genetic DNA (genes) into RNA, each gene can be modeled as a switch that can be “ON” (RNA is being transcribed at a minimal functional level) or “OFF” (RNA is being transcribed below a minimum functional level). The presence of RNA transcribed by a gene can launch a process that eventually can inhibit or promote the production of RNA by other genes, in the fashion of a boolean logical circuit.

**Fig. 1:**
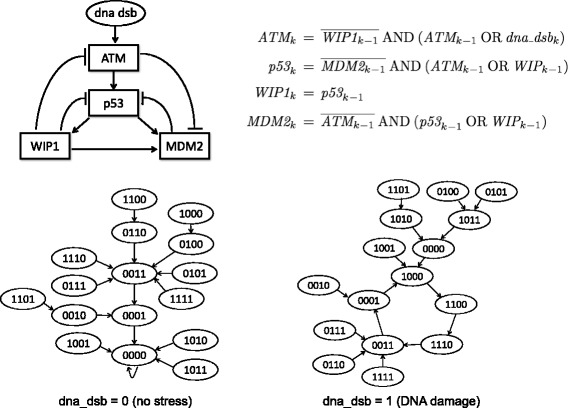
The p53-MDM2 Boolean gene regulatory network. The state of the system at time *k* is represented by a vector (*ATM*_*k*_,*p53*_*k*_,*WIP1*_*k*_,*MDM2*_*k*_), where the subscript *k* indicates expression state at time *k*. The Boolean input *u*_*k*_=*dna_dsb*_*k*_ at time *k* indicates the presence of DNA double strand breaks. Counter-clockwise from the top right: the activation/inhibition pathway diagram, transition diagrams corresponding to a constant inputs *dna_dsb*_*k*_≡0 (no stress) and *dna_dsb*_*k*_≡1 (DNA damage), and Boolean equations that describe the state transitions

Figure 1 depicts an example of a Boolean network model of a gene regulatory network. This is the *p53*-*MDM2* negative feedback loop transcriptional circuit that is involved in DNA repair in the cell, and is therefore an important tumor suppression agent [8]. The diagram in the top left displays the activation/inhibition pathways corresponding to this gene regulatory network. In the upper right, we see Boolean equations consistent with the pathway diagram, which specify the associated Boolean network [9]. From the pathway diagram, is is clear that *MDM2* has an inhibiting effect on *p53*, which in turn activates it. This *p53*-*MDM2* negative-feedback regulatory loop keeps *p53* at small expression levels under no stress, in which case all four proteins are inactivated in the steady state [8]. However, *MDM2* is also inhibited by *ATM*, which in turn is activated by the DNA damage signal, so that *p53* is expected to display an oscillatory behavior under DNA damage [10]. These behaviors are captured nicely by the BN model, as can be seen in the state transition diagram under no stress and under DNA damage, at the bottom of Fig. 1.

The basic issue with the Boolean network model is that it is deterministic and thus unable to cope with uncertainty due to noise and unmodeled variables. Stochastic models have been proposed to address this, including Random Boolean Networks [11], Boolean Networks with perturbation (BNp) [12], and Probabilistic Boolean Networks (PBN) [13]. The R package *BoolNet* [14] implements the BN and PBN models, including asynchronous and temporal networks. It provides essential analysis tools and a simple but complete interface for user entry of BN models.

**Fig. 2:**
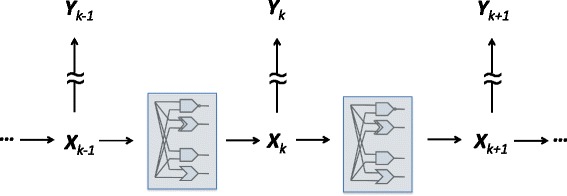
POBDS model. The state process vector **X**_*k*_ evolves through networks of Boolean functions (i.e., logical gates), but it cannot be observed directly; instead, an incomplete and noisy function of the state is observed, namely, the observation process vector **Y**

A key point is that all aforementioned models assume that the Boolean states of the system are directly observable. But, in practice, this is never the case. Modern transcriptional studies are based on technologies that produce noisy indirect measurements of gene activity, such as cDNA microarrays [15], RNA-seq [16], and cell imaging-based assays [17]. The Partially-Observed Boolean Dynamical System (POBDS) signal model [18–20] addresses the noisy observational process, as well as incomplete measurements (e.g., some of the genes in a pathway or gene network are not monitored). In the POBDS model, there are two layers or processes: the Boolean network layer, which is a hidden layer, is the state process, while the observation layer or process models the actual data that are available to researchers – see Fig. 2 for an illustration. It should be noted that the POBDS model is a special case of a hidden Markov model (HMM), in which the underlying states are Boolean.

The purpose of the present paper is to describe the *BoolFilter* R package, which implements the POBDS model and associated algorithms. It allows the user to estimate the Boolean states, network topology, and noise parameters from time series of transcriptomic data using exact and approximated (particle) filters, as well as simulate the transcriptomic data for a given Boolean network model. Some of its infrastructure, such as the network interface, is the same as in the *BoolNet* package. This enhances compatibility and user accessibility to the new package. The *BoolFilter* package can be considered to be an extension of the *BoolNet* package to accommodate the POBDS model. *BoolFilter* does not replace *BoolNet*, but instead both packages can be used together.

Several tools for the POBDS model have been proposed recently. The optimal estimators based on the MMSE criterion, called the Boolean Kalman Filter (BKF) and Smoother (BKS), were introduced in [21, 22], respectively. In addition, methods for simultaneous state and parameter estimation and their particle filter implementations were developed in [18, 19]. Other tools include optimal filter with correlated observation noise [23], network inference [24], sensor selection [25], fault detection [26], and control [20, 27–29]. *BoolFilter* implements the exact BKF and BKS, an approximate filter based on the SIR particle filtering approach, as well as a multiple model adaptive estimator (MMAE) for network inference and noise estimation. In *BoolFilter*, Boolean networks are defined by the user through the same interface used in the *BoolNet* package.

## Implementation

The first step for using the package is to define the state process, including the Boolean network and its inputs and noise parameters, and the observation process, which is specific to each kind of expression technology used.

### State process

Assume that the system is described by the state process {**X**_*k*_;*k*=0,1,…}, where **X**_*k*_∈{0,1}^*d*^ represents the activation/inactivation state of the genes at time *k*. The states are assumed to be updated and observed at each discrete time through the following nonlinear signal model:

1 $$ \mathbf{X}_{k} = \mathbf{f}\left(\mathbf{X}_{k-1}\right) \,\oplus\, \mathbf{n}_{k} \,\quad \text{(state model)},   $$

for *k*=1,2,…. Here, **n**_*k*_∈{0,1}^*d*^ is the transition noise at time *k*, “ ⊕” indicates component-wise modulo-2 addition, **f**:{0,1}^*d*^→{0,1}^*d*^ is the *network function*. The noise process {**n**_*k*_;*k*=1,2,…} is assumed to be independent, meaning that the noises at distinct time points are independent random variables, and it is also assumed that they are independent of the initial state **X**_0_. In addition, **n**_*k*_ is assumed to have independent components distributed as Bernoulli(*p*) random variables, where the noise parameter *p* gives the amount of “perturbation” to the Boolean state transition process. As *p*→0.5, the system will become more and more chaotic, however as *p*→0 the state trajectories become more deterministic and therefore become governed more tightly by the network function.

The network function specifies the Boolean network. In the *BoolFilter* package, the network function is entered using the *BoolNet* package vernacular. The user can define their own Boolean Network using the *BoolNet* function *loadNetwork*, or use the available predefined networks. In addition to the “cellcycle” network, defined in the *BoolNet* package, *BoolFilter* contains three additional well-known and frequently researched networks in its database: “p53_DNAdsb0”, “p53_DNAdsb1”, and “Melanoma”. Notice that “p53net_DNAdsb0” and “p53net_DNAdsb1” refer to the p53-Mdm2 negative feedback loop regulatory network with external input 0 and 1 respectively, shown in Fig. 1. For example, the *p53net_DNAdsb1* network can be called as follows:



For more information about defining a custom Boolean network using the *BoolNet* interface, the reader can refer the *BoolNet* package documentation [14].

### Observation model

Several common observation models are implemented in the package:

- Bernoulli Model: the observations are modeled to be the perturbed version of Boolean variables with i.i.d. Bernoulli observation noise with intensity *q*. This is defined in *BoolFilter* as:

- Gaussian Model: the Boolean variables in inactivated and activated states are assumed to be observed through Gaussian distributions ${\mathcal N}(mu0,sigma0)$ and ${\mathcal N}(mu1,sigma1)$ respectively, where (*mu*0,*sigma*0) and (*mu*1,*sigma*1) are the means and standard deviations of the observed variables in the inactivated and activated states, respectively. The Gaussian model is appropriate for important gene-expression measurement technologies, such as cDNA microarrays [30] and live cell imaging-based assays [31], in which measurements are continuous-varying and unimodal (within a single population of interest). More information about this model can be obtained in [20]. For example,

- Poisson Model: a common model for RNA-Seq data, which has the following parameters: sequencing depth *s*, baseline expression in inactivated state *mu*, and the differential expression *delta* between activated and inactivated expression levels, which is a vector of size *d*. More information about this model can be obtained in [22, 26, 32]. For example,

- Negative Binomial Model: another common model for RNA-Seq data, which, in comparison with the Poisson model, carries an extra parameter vector *phi*. This is called the inverse dispersion parameter and regulates the variability in the measurement independently of the mean. More information about this model can be obtained in [29]. For example,

### Data generation - *simulateNetwork()*

After defining the state and observation models, the user is able to create a time series of state and observation data of a user-defined size *n*.*data* as follows:



where *obsModel* specifies the observation noise model, as described in the previous section.

### Boolean Kalman filter - *BKF()*

The BKF [21] is the optimal recursive MMSE state estimator for a partially-observed Boolean dynamical system. The BKF can be invoked as follows:



where *Y* is the observation data subset from the output of the *simulateNetwork()* function (here called *data$Y*) or a set of real observation data, and *p* is the magnitude of the state transition noise.

### Particle filter approximation of BKF - *SIR_BKF()*

When the network contains many nodes, i.e., genes, the exact computation of the BKF becomes intractable, due to the large size of the matrices involved. Other methods, such as sequential Monte-Carlo methods (also known as particle filtering algorithms), must be used to approximate the optimal solution. The particle filter implementation of the BKF (based on sequential importance resampling (SIR)), called *SIR_BKF* [33], can be applied as follows:



where *N* is the number of particles and 0≤*alpha* ≤ 1 is a threshold for the particle-filter resampling process. For more information, see [33].

### Boolean Kalman smoother - *BKS()*

The BKF uses the latest measurement **Y**_*k*_ to estimate the state at the current time *k*. Assume, however, that one wants to use the entire **Y**_1:*k*_ data sequence to estimate the state at a current or a previous instant of time *s*, where 1<*s*<*k*. This would be the case if data have been collected and stored “off-line” up to time *k*, and estimates of the states at a time *s*, where *s*<*k*, are desired. The Boolean Kalman Smoother (BKS) [22] is the optimal MMSE smoother in this case. This estimator can be called as follows:



### Multiple model adaptive estimator - *MMAE()*

Suppose that the nonlinear signal model is incompletely specified. For example, the topology (i.e., the connections) of the Boolean network may be only partially known, or the statistics of the noise processes may need to be estimated. We assume that the information to be estimated can be coded into a vector parameter *Θ*={*θ*_1_,…,*θ*_*M*_}. Model selection is achieved by running a bank of BKFs running in parallel, one for each candidate model. The following two cases can currently be handled by the *MMAE* function:

- Unknown network: The user can define multiple networks as possible models of the system.
- Unknown process noise: Different possible intensities of Bernoulli process noise can be entered as an input to the function.

An example of implementation of algorithm, when there are two possible models for the network (*“net1”, “net2”*) and three possibilities of process noise, is as follows:



where *Prior* is a vector of size |*net*|×|*p*| that specifies the probability of each model (if Prior is defined as “NA”, the uniform prior is assumed as a default for different models), and “threshold” stops algorithm when the posterior probability of any model surpass this value.

### Plotting trajectories - *plotTrajectory()*

A sequence of data can be plotted using the *plotTrajectory* function. This function can be used for plotting the sequence of state, observations, or estimation results - or any combination of the above. The user is able to specify the Boolean variables which should be plotted. For example,



where *data$X* and *Results$Xhat* are the original and estimated sequence of states, respectively.

## Results and discussion

**Fig. 3:**
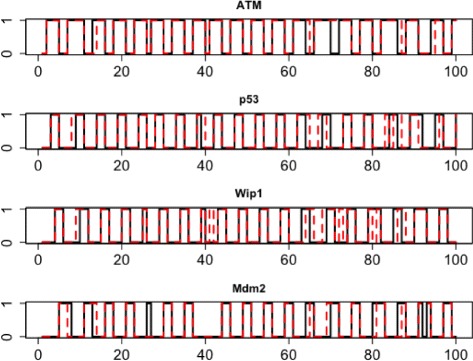
Typical graphical output of the function *“plotTrajectory”*. The black and red lines denote the original state trajectory and estimated trajectories by the BKF for all four genes

We provide below a detailed step-by-step demonstration of a typical *BoolFilter* session. First, the p53-MDM2 Boolean network model in both no-stress (*dna_dsb=0*) and DNA-damage (*dna_dsb=1*) conditions is loaded as:



We assume a POBDS observation model that consists of Gaussian gene expression measurements at each time point:



To evaluate the performance of the BKF in estimating the gene states, a time series of length 100 time points is generated from the “p53net_DNAdsb1” network, with Bernoulli process noise of intensity *p*=0.01:



The BKF can be called for estimation purposes as follows:



The result can be visualized using the *plotTrajectory()* function as:



Figure 3 displays the obtained plots, showing the original and estimated transcriptional states of all four genes over the length of the time series. The solid black lines specify the original gene trajectories and the dashed red lines correspond to the trajectories estimated by the BKF. One can see that the states of all genes are properly tracked by the BKF.

**Fig. 4:**
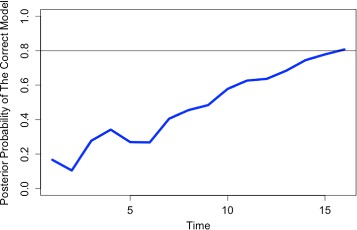
Typical graphical output of the function *“MMAE”*. The Multiple-Model Adaptive Estimation algorithm determines which input network is the most likely network and creates a graphical output of the posterior probability of the selected model

To evaluate the performance of the *MMAE()* function for system identification purposes, the “p53net_DNAdsb” networks are used with a Gaussian observation model. We assume that the network in which the data is generated from and also the intensity of the process noise (*p*) are unknown. The network function is assumed to be either “p53net_DNAdsb0” or “p53net_DNAdsb1”, and the intensity of process noise is assumed to be one of *p*=0.01, *p*=0.05 or *p*=0.10. Thus, there are 6 possible models for the system. A uniform prior assumption is considered for all models. The *MMAE()* function can be performed for model selection purposes as follows:



The stopping threshold for the *MMAE()* function is set to be 0.8. This means that if the posterior probability of any model exceeds this value, the decision is made and the process is stopped (for more information see [24]). Figure 4 displays the posterior probability of the true model. It can be seen that after only 15 time points, the true model is found by the MMAE method and the process is stopped. Future versions of the *BoolFilter* package will include methods for simultaneous estimation of state and parameters of POBDS as well as optimal control algorithms for modifying the behavior of the system (e.g. reducing the probability of states associated with cancer in the gene regulatory example).

## Conclusion

The *BoolFilter* R package enables estimation and identification of gene regulatory networks observed indirectly through noisy measurements based on various expression technologies such as cDNA micrroarys, RNA-Seq, and cell imaging-based assay. This software tool provides the bioinformatics community with state-of-the-art exact and approximate algorithms for estimation of transcriptional states of genes in small and large systems, respectively, as well as identification of gene regulatory networks, which is a key step in the investigation of genetic diseases. The performance of the software in estimation and identification of gene regulatory networks was demonstrated using the p53-MDM2 Boolean gene regulatory network.

## Availability and requirements

**Project name:** BoolFilter Package

**Project home page:** https://cran.r-project.org/web/packages/BoolFilter/index.html

**Operating system(s):** Platform independent

**Programming language:** R

**License:** GNU GPL

**Any restrictions to use by non-academics:** none

## Abbreviations

BKF: Boolean Kalman filter
BKS: Boolean Kalman smoother
BN: Boolean network
MMAE: Multiple model adaptive estimator
POBDS: Partially-observed Boolean dynamical system
SIR: Sequential importance resampling
